# Value of intra-tumor heterogeneity evaluated by diffusion-weighted MRI for predicting pathological stages and therapeutic responses to chemoradiotherapy in lower rectal cancer

**DOI:** 10.7150/jca.38354

**Published:** 2020-01-01

**Authors:** Michihiro Kudou, Masayoshi Nakanishi, Yoshiaki Kuriu, Yasutoshi Murayama, Tomohiro Arita, Mitsuo Kishimoto, Eiichi Konishi, Mariko Goto, Kei Yamada, Eigo Otsuji

**Affiliations:** 1 Division of Digestive Surgery, Department of Surgery, Kyoto Prefectural University of Medicine, Kyoto, Japan;; 2 Department of Surgical Pathology, Kyoto Prefectural University of Medicine, Graduate School of Medical Science, Kyoto, Japan;; 3 Department of Radiology, Graduate School of Medical Science, Kyoto Prefectural University of Medicine, Kyoto, Japan.

**Keywords:** rectal cancer, chemoradiotherapy, magnetic resonance imaging, diffusion-weighted image, radiomics.

## Abstract

**Aim:** Diffusion-weighted MRI (DWI) has the potential to reveal intra-tumor structural heterogeneity consisting of stroma through an evaluation of uniformity on DWI. In present study, we examined the diagnostic value of intra-tumor heterogeneity evaluated by DWI in lower rectal cancer (LRC).

**Patients and Methods:** A total of 172 LRC patients underwent radical surgery between 2009 and 2017. T1 tumors and cases without pre-operative MRI were excluded. Twenty-nine primary resection cases (PR) and 37 pre-operative chemoradiotherapy followed by radical surgery cases (pCRT) were targeted. Intra-tumor heterogeneity on DWI was quantified using a specific formula (HSD). Structural heterogeneity was objectively quantified by an image analysis of resected specimens using a digital microscope (HSP). The relationships between HSD and HSP, pathological factors, and tumor regression grades (TRG) of pCRT were evaluated.

**Results:** The relationship between HSD and HSP was analyzed by a linear regression model in PR cases, revealing a positive correlation (R^2^=0.43). PR cases were divided into HSD-high and HSD-low according to the median. There were more pT3 or N(+) cases in HSD-high (*p*=0.038, 0.095). HSD before pCRT correlated with TRG (grade 1 versus 2/3) in pCRT cases (*p*=0.001). The diagnostic accuracy of HSD for predicting T and N stages and therapeutic grades was evaluated by cut-off values calculated using a ROC curve and revealed that each factor may be accurately diagnosed.

**Conclusion:** Intra-tumor heterogeneity on DWI corresponded with stromal pathological heterogeneity. It is useful for predicting T3 or deeper tumor invasion, pathological N(+), and the therapeutic effects of pCRT.

## Introduction

Recent advances in treatments directed at lower rectal carcinoma (LRC) have resulted in multimodal therapies with curative potential and the preservation of quality of life. Pre-operative chemoradiotherapies (pCRT) followed by radical surgery are attracting increasing attention worldwide^1^-^3^. Previous studies reported that radical surgery after pCRT for LRC contributed to anal preservation, reduced the rate of local recurrence, and secured a circumferential resection margin^4^-^7^. Furthermore, the “watch and wait” was proposed by Habr-Gama A et al, which represented an approach in the management of clinical complete responses after pCRT for LRC^8^. This approach was validated in many studies, revealing favorable findings for oncological outcomes^9^, ^10^.

Total mesorectal excision (TME) with lymph node dissection (LND) is the standard surgery for LRC^11^-^13^. After Heald. R. J. initially proposed the concept of TME, the importance of this surgical method was repeatedly demonstrated worldwide. However, the area of LND for LRC remains controversial. Two routes of lymphatic flow around the lower rectum generally exist: upper flow along the superior rectal artery and towards the inferior mesenteric artery, and lateral flow along the middle and inferior rectal arteries and towards the internal iliac artery. A previous study reported that lymph node metastases (LNM) through lateral lymphatic flow were observed in 20.1% of LRC, which invaded the surrounding tissue deeper than the muscularis propria, or in 27% of LRC with LNM in the mesorectum^14^. Therefore, lateral LND (LLND) in addition to mesorectal lymph nodes for such advanced LRC is recommended in Japan^15.^ A Japanese randomized control trial (JCOG0212) that compared oncological outcomes between TME alone and TME plus LLND actually revealed that LLND significantly decreased the rate of local recurrence. However, some complications are associated with LLND; the incidence rates of urinary disturbance and sexual function disorders after LLND are high. Therefore, indications for the omission of LLND after pCRT have also been investigated in Japan^16^,^17^.

Accurate diagnostic modalities are essential for deciding the indication for LLND, the “watch and wait” approach, and diagnosing clinical responses to pCRT. Several pre-operative examinations are generally performed to evaluate LRC patients, such as colonoscopy, biopsy, contrast-enhanced computed tomography (CT), positron emission tomography/CT, and magnetic resonance imaging (MRI). In these modalities, the efficacy of the diffusion-weighted images (DWI) of Magnetic Resonance Imaging (MRI) has been actively investigated and reported. DWI show the diffusional restriction of water movement in stromal areas of tissue as pixel values. The structure of the stromal area in solid tumors is more complex than that in normal tissues due to the proliferation and invasion of cancer cells and the formation of cancer-associated stroma, resulting in the restriction of water movement inside tumor tissue. Therefore, tumor tissue shows higher intensity signals than normal tissue on DWI, which contributes to the clinical diagnosis of tumors^18^. The high accuracy of DWI for evaluating the persistence of tumors after pCRT was previously demonstrated and is now widely used in clinical settings^19^-^21^. The involved and heterogeneous structure consisting of stoma is formed in tumors with strong infiltration; furthermore, the survival of colon cancer patients with these tumors was previously reported to be poor^22^, ^23^. Due to the superiority of DWI for evaluating stroma, the complexes and heterogeneities of stromal structures may be consistent with the intra-tumor heterogeneity of intensity on DWI. Hence, we hypothesized that these stromal or structural complexes and heterogeneities may be indirectly evaluated using DWI, and it may be similarly useful to predict malignant potential of LRC.

In the present study, intratumor heterogeneity of LRC on DWI image was quantified using unique formula (Heterogeneous score of DWI: HSD). Moreover, intratumor stromal heterogeneity in pathological findings was similarly quantified using objective image analysis that was performed using a digital microscope, software, and unique formula (Heterogeneous score of pathology: HSP). To validate the hypothesis, the association of HSD with HSP was statistically analyzed. Subsequently, the value of HSD in clinical settings was investigated through a retrospective analysis of LRC patients who underwent radical surgery alone and pre-operative CRT followed by radical surgery. The present study aimed to investigate whether intratumor stromal heterogeneity could be predicted using image analysis of DWI, and to clarify the clinical value of intra-tumor heterogeneity on DWI in LRC.

## Patients and methods

### Study design and patients

**Figure 1:**
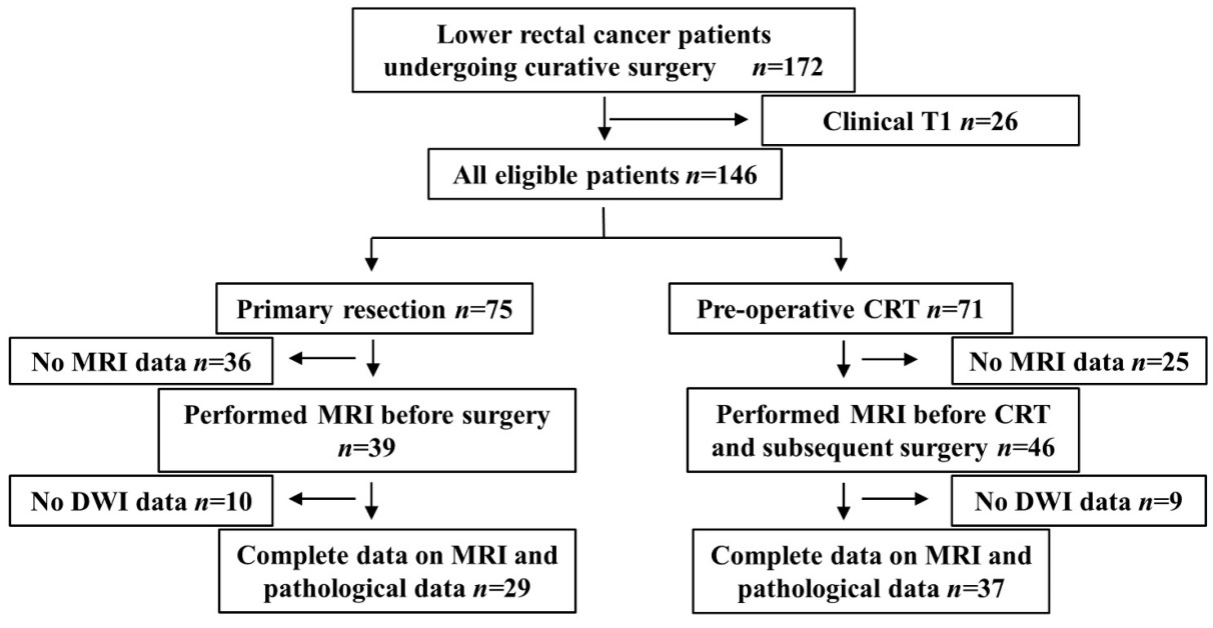
Flow chart showing the selection process for patient inclusion in the present study.

This was a respective diagnostic accuracy study in order to investigate the diagnostic value of intra-tumor heterogeneity evaluated by DWI in lower rectal cancer. The present study included LRC patients with tumors that were mainly located under the peritoneal reflection, which was evaluated by lateral image of contrast enema examination, who underwent radical surgery with regional LND between April 2009 and March 2017 at the Division of Digestive Surgery of Kyoto Prefectural University of Medicine (KPUM). Clinically diagnosed T1 cases were excluded because of the difficulties associated with detecting the primary tumor by MRI. One-hundred and forty-six cases fulfilled this criterion, 75 of which underwent primary resection, and the remaining 71 underwent pCRT followed by radical surgery. Cases without MRI containing DWI images before surgery and pCRT were excluded. Therefore, 29 primary resection cases (primary resection group) and 37 undergoing pCRT followed by radical surgery (pCRT group) were retrospectively analyzed (Figure 1).

### pCRT followed by surgery

Between 2009 and 2017, two regimens of combined chemotherapies with radiotherapy (RT) were performed in KPUM. The first regime was combination therapy of 80-120 mg/day tegafur, gimeracil, and oteracil potassium (TS-1) and 80 mg/m^2^ irinotecan (CPT-11) (TS-1+CPT-11). TS-1+CPT-11 was routinely used between 2009 and 2015 for LRC cases. The second regime was 80-120 mg/day TS-1 monotherapy between 2016 and 2017. Long-course RT (45 Gy/25 Fr) combined with these chemotherapies was performed.

### Surgical procedures and pathological findings

All surgeries were performed or supervised by surgeons with sufficient experience of rectal resection. The surgical method was selected according to the Japanese colorectal cancer guidelines^16^. LLND was performed against LRC which were clinically diagnosed as T3 or deeper, or LMN.

Resected specimens were microscopically examined by at least two experienced pathologists, and evaluated according to the Japanese Classification System^24^. Briefly, lymph nodes in mesorectum, lateral lymph nodes, and lymph nodes around inferior mesenteric artery were defined as regional lymph nodes of lower rectal cancer. The tumor grade was classified according to the differentiated type which was mainly contained within tumor. The tumor regression grade (TRG) of pCRT was defined as follows according to the Japanese Classification System. Grade 0: no evidence of the tumor ever being treated, Grade 1: regression of less than two-thirds of the tumor, Grade 2: regression of more than two-thirds of the tumor, Grade 3: complete regression.

### Evaluation of intra-tumor heterogeneity by MRI and the quantification of intratumor heterogeneity on DWI image

**Figure 2:**
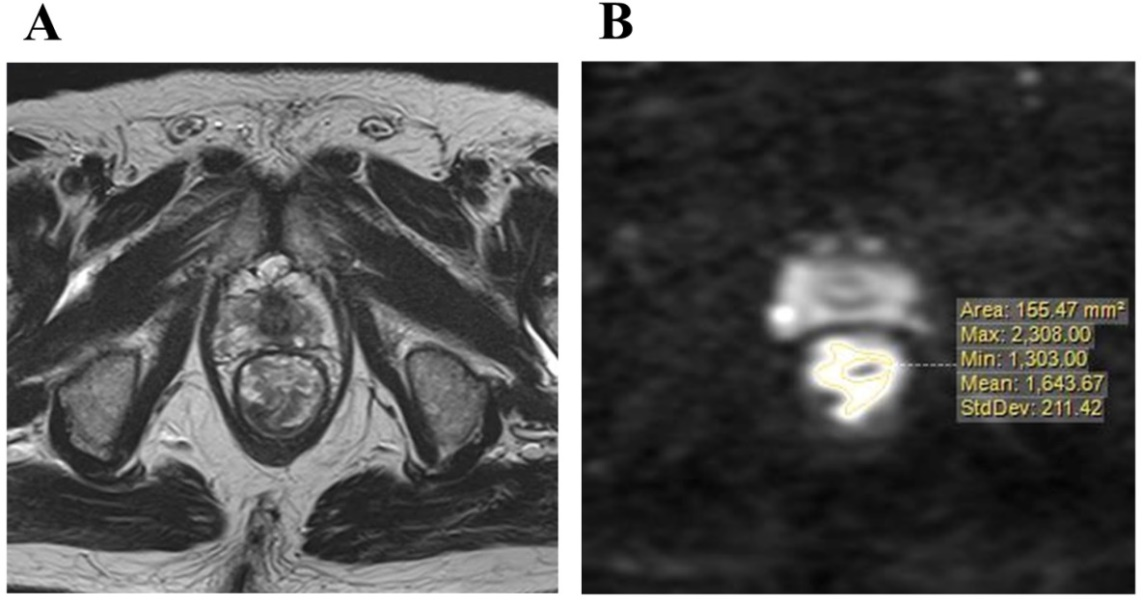
** A:** T2-weighted axial image of the maximum cut surface of lower rectal carcinoma, **B:** The distribution signal intensity on a diffusion-weighted image in the maximum cut surface was measured.

Imaging was performed with a 1.5 or 3.0 T pelvic MRI with pelvic phased-array coils at KPUM or related medical centers. T2-weighted axial images with a section thickness of 5-7 mm and sagittal images with fast spin-echo sequences were acquired. An axial diffusion-weighted sequence with background body signal suppression (DWIBS, b-values 800-1000 s/mm^2^) was also obtained. Primary resection cases underwent MRI for staging before surgery, and pCRT cases for pre-treatment staging and re-staging in order to diagnose therapeutic responses almost 4 to 7 weeks after the completion of pCRT. DWI were evaluated for the maximum cut surface of an axial image of the rectal tumor, which was identified by a T2-weighted axial image. Areas of a higher signal intensity than the normal bowel wall or background on DWI were considered to be the primary tumor. The distribution of signal intensity in this high-intensity area was evaluated, and then maximum (MAX) and minimum (MIN) value of signal intensity in tumor were measured (Figure 2A, B). Furthermore, the intra-tumor heterogeneity of the signal intensity on DWI was quantified using the following formula.

HSD = [(MAX value of signal intensity) - (MIN value of signal intensity)] / [(MAX value of signal intensity) + (MIN value of signal intensity)].

This formula was referred by the NEMA criteria, and was used to evaluate the uniformity of MRI^25^.

### Evaluation of morphological heterogeneity by pathological findings and the quantification of intratumor stromal heterogeneity on pathological image

**Figure 3:**
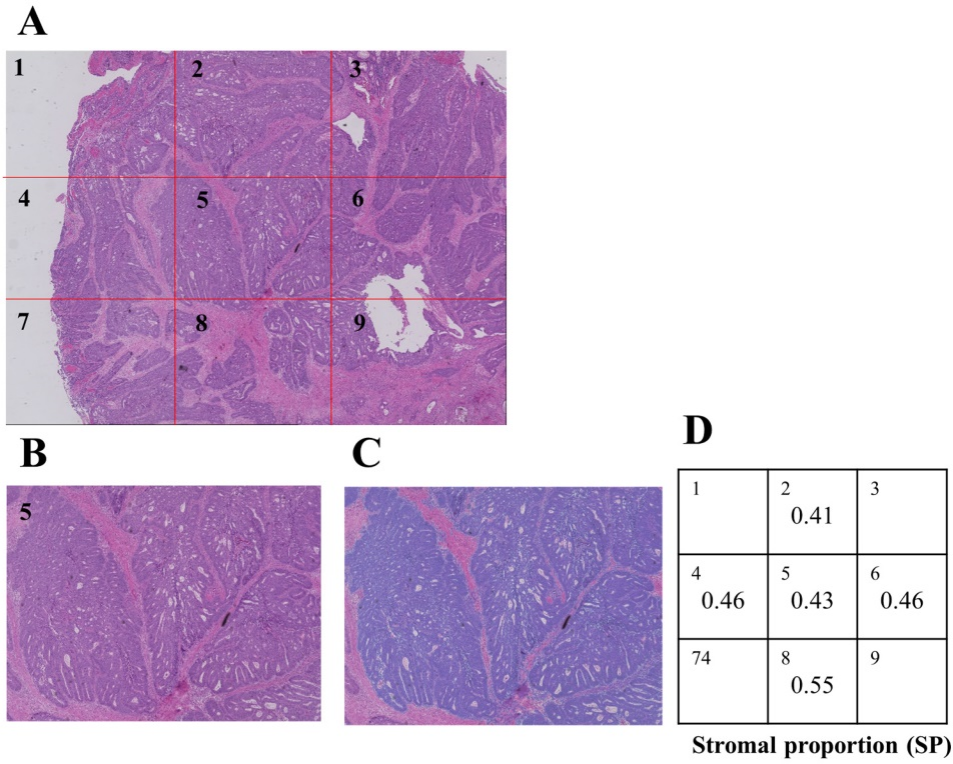
** A:** Combined image of Nine ×40 Hematoxylin-Eosin-stained LRC pictures that were captured and composed using the digital microscope, KEYENCE BZX-700. **B, C:** Cancer cells were masked using the hybrid cell count of specific software (blue color), and the proportion of stroma on images was calculated. **D:** Five out of 9 images were extracted, and morphological heterogeneity was calculated using a unique formula.

Formalin-fixed paraffin-embedded samples were sliced at the maximum cut surface by expert technicians, re-fixed on slide glasses, and stained using Hematoxylin-Eosin. Stained samples were captured using a specific digital microscope (KEYENCE BZ700) (Figure 3A), and the area of cancer cells on ×40 microscopic images was subsequently measured using the hybrid cell count function of KEYENCE software (Figure 3B, C). The proportion of the stromal area on ×40 images was calculated as follows.

[Stromal proportion (SP)] = [(Area of whole tumor tissue) - (Area of cancer cells)] / [(Area of whole tumor tissue)].

The SP of the tumor was measured on 5 different images, and HSP was calculated using the following formula (Figure 3D).

HSP = [(MAX value of SP) - (MIN value of SP)] / [(MAX value of SP) + (MIN value of SP)]

### Statistical Analysis

Comparisons were performed between both groups using the Mann-Whitney U test or Fisher's exact test. *p* values of less than 0.05 were regarded as significant. We performed linear regression analyses to evaluate the relationship between HSD and HSP. Receiver operating characteristic (ROC) curves were generated to evaluate the diagnostic performance of HSD for pre-operative staging and predicting responses to pCRT. The area under the curve (AUC), corresponding values under the ROC curve, sensitivities, specificities, and accuracies were calculated. Statistical analyses were performed using JMP version 10.

## Results

### Clinicopathological characteristics of eligible patients

Patient characteristics were summarized in Table 1. MRI for re-staging was performed 21-61 days (median: 33 days) after pCRT in pCRT group. Thirty-three cases of them (89.1%) underwent MRI between 4 and 7 weeks after pCRT. LNM was clinically diagnosed as positive in 12 cases of primary resection and confirmed by pathological findings. Although LNM was clinically diagnosed in 26 cases of pCRT, only 16 were pathologically diagnosed with LNM. In primary resection cases, tumor invasion depth was pathological T2 in 7 cases (24.1%) and T3 in 22 (75.8%). In pCRT cases, tumor invasion depth was pathological T1 in 2 cases (5.4%), T2 in 9 cases (24.3%), T3 in 23 cases (62.1%), and T4 in 3 cases (43.2%). The pathological TRG of pCRT was grade 1 in 14 cases (37.8%), grade 2 in 21 (56.7%), and grade 3 in 2 (5.4%).

### Relationship between intra-tumor heterogeneity on DWI and structural heterogeneity in pathological findings

**Figure 4:**
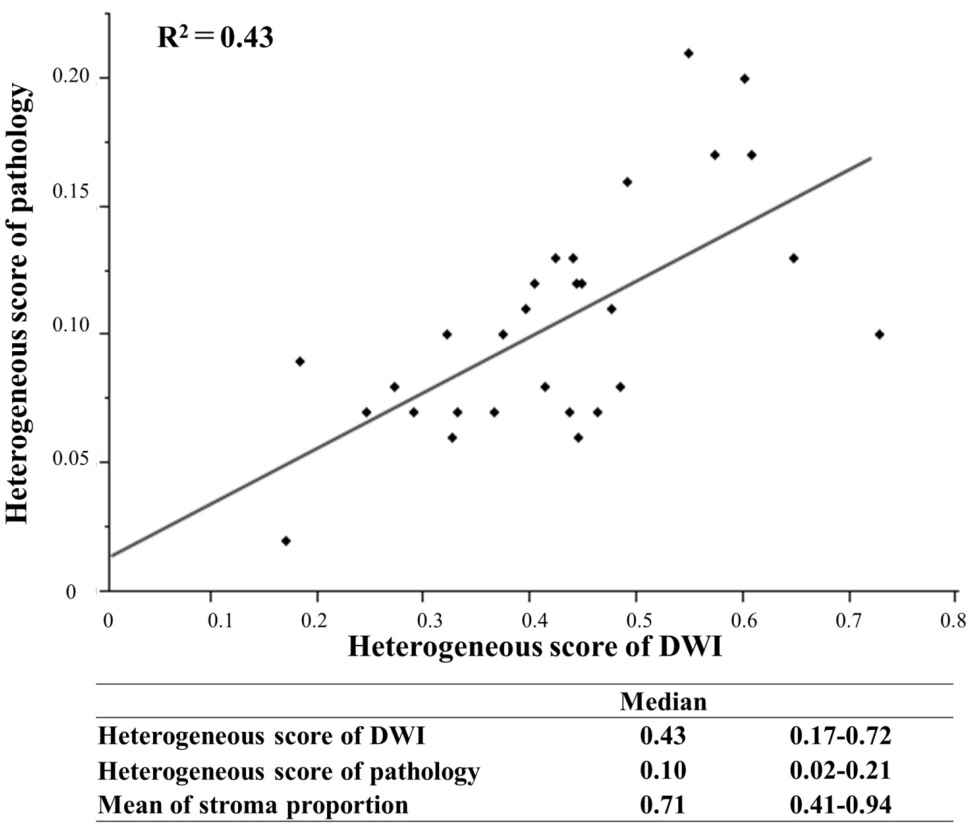
Linear regression model to evaluate the relationship between the heterogeneous score of DWI and pathology.

The relationship between HSD and HSP was evaluated in primary resection cases, in which tumor tissue was not affected by some treatment. In pathological findings, the mean proportion of the stroma in tumor tissue was 0.41-0.94 (median: 0.71). HSP was 0.02-0.21 (median: 0.10), and HSD was 0.17-0.72 (median: 0.43) with a higher score reflecting a more complex structure. A linear regression model, which was used to evaluate the relationship between stromal structural heterogeneity and the intra-tumor heterogeneity of signal intensities on DWI, revealed a positive correlation (R^2^=0.43) (Figure 4).

### Relationships between HSD and pathological factors in the primary resection group

Primary resection cases were divided into two groups according to the median value of HSD: HSD-high (HSD>0.43) and HSD-low (HSD ≤0.43). The relationships between HSD and pathological factors were statistically analyzed (Table 2). The number of T3 tumors was significantly higher in HSD-high (*p*=0.038), and the number of tumors with LNM was slightly higher in HSD-high (*p*=0.095).

### The value of HSD for predicting the therapeutic grade of pCRT

**Figure 5:**
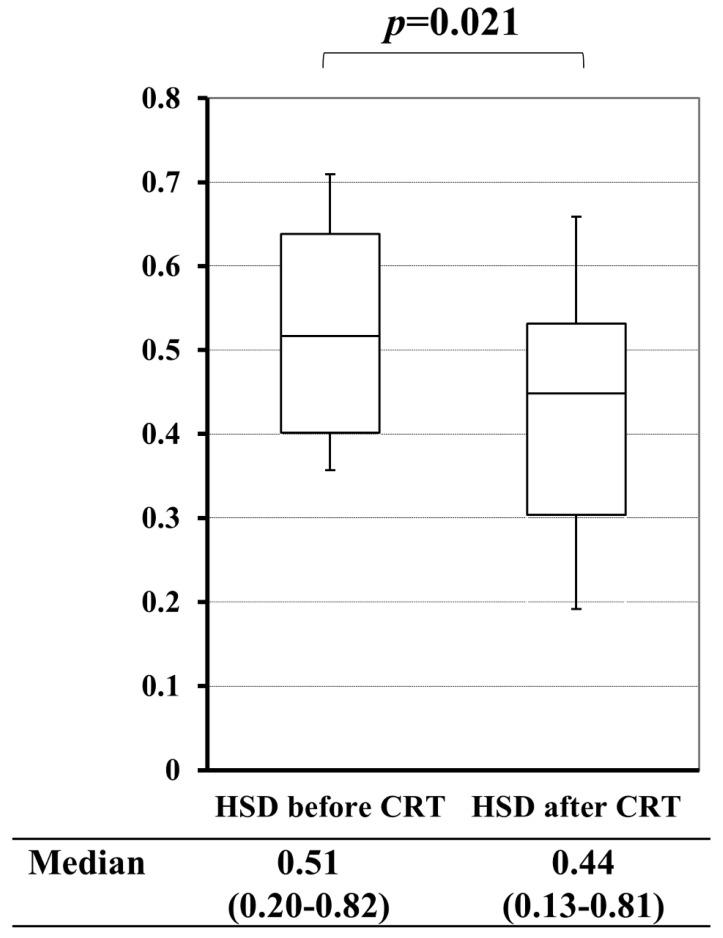
A comparison of the heterogeneous score of diffusion-weighted images (HSD) before and after chemoradiotherapy (CRT).

In pCRT cases, a comparison between HSD before and after pCRT revealed that pCRT significantly decreased HSD of the primary tumor (*p*=0.021) (Figure 5). Based on these results, we investigated the value of HSD for predicting the therapeutic grade of pCRT. Mean HSD before pCRT for the tumor, for which the TRG of pCRT were 1/2 and 3, were 0.63 and 0.46, respectively. This difference was significant (*p*=0.001) (Table 3).

### Diagnostic accuracy for predicting pathological staging and therapeutic grades of pCRT

To evaluate the diagnostic accuracy of HSD, a ROC curve was used to select the cut-off value for predicting T (T3 vs T2) and N staging (N+ vs N-) as well as TRG of pCRT (grade 1 vs 2/3). The AUC values of the ROC curve to predict T, N staging, and TRG were 0.792, 0.725, and 0.801, respectively. The most appropriate cut-off values according to the ROC curve for predicting T and N staging as well as the therapeutic grade were 0.445, 0.448, and 0.695, respectively. T stage (T3) was diagnosed with high specificity (100%) and a positive predictive value (PPV, 100%), as was the N stage (N+) with high specificity (82.4%) and accuracy (86.2%) in primary resection cases. The therapeutic grade of pCRT (Grade 1) was diagnosed with high specificity (95.8%) and accuracy (97.3%) (Table 4).

## Discussion

The relationship between stroma around cancer cells and malignant potential has been attracting increasing attention. Morphological structures, molecular expression, and cancer-associated fibroblasts in stoma were shown to be related to survival, recurrence, and responses to some chemotherapies and radiotherapies^26^-^28^. The tumor-stroma ratio (TSR) is recognized as a useful index to evaluate stromal distribution in tumors and predict oncological outcomes^29^,^30^. However, some issues are associated with the use of TSR in clinical settings. First, such pathological biomarker cannot be used in pre-operative setting because fully resected specimens of tumors are needed. Second, structural and morphological heterogeneities, which consist of stroma and cancer nests, cannot be evaluated using this method. Third, intraobserver and interobserver differences may occur in the diagnosis of TSR. To overcome these issues, we evaluated a relatively large area of cancerous tissue using DWI; furthermore, intra-tumor heterogeneity was indirectly quantified through an image analysis of DWI using a unique formula in pre-operative settings. TSR was evaluated using an objective method, digital microscope, and specific software. To the best of our knowledge, the present study, the results of which showed a relationship between the intra-tumor heterogeneity of tumors on DWI and cancerous stroma evaluated by pathological findings of LRC using objective methods (Figure 4), is the first report worldwide.

Based on the results obtained herein, we hypothesized that intra-tumor heterogeneity on DWI may be associated with malignant potential, e.g. tumor size, T and N stages, and ductal invasion, as reported in previous studies using pathological specimens. The present study revealed that the number of T3 tumors was significantly higher, while the number of LNM cases was slightly higher among highly heterogeneous tumors on DWI in the primary resection group (Table 2), suggesting that HSD is a predictor of T and N stages. The specificity and PPV for predicting T3 or deeper using the cut-off value of HSD, which was calculated by the ROC curve, were 100.0 and 100.0%, respectively. The specificity and PPV for predicting LNM were 82.4 and 72.7%, respectively (Table 4). These are regarded as the risk factors of lateral LNM^14^. Therefore, HSD may be useful to decide the indication of LLND by the prediction of T3 or LNM. The diagnostic accuracies of T and N stages in LRC were not satisfactory using conventional methods, resulting in the over-indication of LLND. The JCOG0212 trial also reported that lateral LNM was positive in only 7% of TME with LLND cases; furthermore, pathologically-diagnosed T stages were T1 and T2 in 26% of TME with LLND, indicating that LLND may not be necessary for these cases^15^, ^31^. Our criteria using HSD are expected to reduce the over-indication of LLND.

The efficacy of the area or volume with high intensity on DWI for diagnosing the therapeutic effects of CRT in LRC was previously reported. Therefore, we investigated the value of HSD for predicting therapeutic responses to CRT. CRT significantly suppressed intra-tumor heterogeneity on DWI (*p*=0.021) (Figure 5). It was assumed that the homogenization of the intra-tumor morphological structure was due to tumor shrinkage or fibrosis. The area of a tumor with a high intensity signal on DWI before or after pCRT correlated with TRG, grade 1 vs 2/3, as previously reported^29^ (*p*=0.001, *p*<0.001, respectively) (data was not shown). HSD before pCRT correlated with TRG (*p*=0.001), and TRG (grade 1) was predicted with high sensitivity and accuracy using the cut-off value of HSD (Table 3,4), suggesting that HSD is imaging biomarker for predicting of TRG.

There were some limitations that need to be addressed. This was a retrospective study with a small sample size, which may limit the statistical power and generate a statistical bias. Further validation may be needed by prospective study with large sample size to prove our hypothesis. Although DWI data need to be collected using the same type of MRI and protocol, it was not possible to unify our data because they were obtained from multiple hospitals. DWI are affected by the background on T2-weighted images, which is known as “T2 shine-though” 33,34. It is often observed in LRC containing mucinous components as showing very high intensity on T2-weighted images. To avoid such interference with the results obtained, an apparent diffusion coefficient map (ADC) was used. However, ADC were not obtained because the ADC was not routinely made. The accuracy of the quantification of heterogeneity may be higher using a statistical method, such as texture analysis, than our formula. However, a simple method needs to be used in clinical settings; thus, we applied this to the present study. Despite these limitations, the present study revealed a relationship between intra-tumor heterogeneity on DWI and structural heterogeneity consisting of stroma, and showed that this intra-tumor heterogeneity on DWI, which may be evaluated in pre-operative settings, was imaging biomarker for predicting pathological T ,N stages, and TRG of pCRT. Although the present results need to be validated in studies with large sample sizes, they will contribute to improvements in the clinical outcomes of LRC.

## Conclusion

In consideration of many limitations such as small sample size, the quantification of intra-tumor heterogeneity on DWI may corresponded to structural heterogeneity consisting of stroma in pathological findings. In consideration of many limitations, it may be useful for predicting T3 or deeper, LNM, which had the potential of a lateral LNM, and the therapeutic effects of pCRT.

## Ethical approval and consent to participate

This study was approved by the Research Ethics Committee of the Kyoto Prefectural University of Medicine (No. ERB-C-1187 and ERB-C-1401). Comprehensive informed consent for use of clinical data and samples was obtained from all eligible patients.

## Figures and Tables

**Table 1:** Patient clinicopathologic characteristics.

		Primary resection group			Preoperative CRT group		
		n=29			n=37		
Gender	Male	20	68.9	%	26	70.2	%
	Female	9	31.0	%	11	29.7	%
Age			64.0±13.5			61.0±9.8	
Duration between MRI and surgery	Median	21	2-70	days			
Duration between CRT and MRI	Median				33	17-61	days
Duration between MRI and surgery	Median				26	8-79	days
Clinical T stage	T2	9	31.0	%	3	8.1	%
	T3	16	55.1	%	27	72.9	%
	T4	4	13.7	%	7	18.9	%
Clinical N stage	Positive	12	41.3	%	26	70.2	%
Pathological tumor size	Mean ± SD		52.6±18.5	mm		34.3±17.4	mm
Main histologic type	Well	20	68.9	%	25	67.5	%
	Moderate	5	17.2	%	4	10.8	%
	Mucinous	3	10.3	%	3	8.1	%
	Signet cell	1	3.4	%	3	8.1	%
Pathological T stage	Scar				2	5.4	%
	T1				2	5.4	%
	T2	7	24.1	%	9	24.3	%
	T3	22	75.8	%	23	62.1	%
	T4				3	8.1	
Pathological N stage	positive	12	41.3	%	16	43.2	%
M stage	positive	4	13.7	%	1	2.7	%
Tumor regression grade after CRT	1				14	37.8	
	2				21	56.7	
	3				2	5.4	

CRT: chemoradiotherapy, MRI: magnetic resonance image, SD: standard deviation.

**Table 2:** The association of heterogeneous score of DWI with pathological factors in primary resection group.

			HSD					
			high (0.43<) (n=14)			low (≤0.43) (n=15)		*p* value
Maximum tumor diameter	Mean ± SD		58.7 ± 18.3	mm		41.1 ± 17.5	mm	0.097
Main histological type	Non-dif	1	7.1	%	3	20.0	%	0.940
Pathological T stage	T2	1	7.1	%	6	40.0	%	0.038
	T3	13	92.8	%	9	60.0	%	
Lymph node metastasis	Positive	8	57.1	%	4	26.7	%	0.095
Lymph ductal invasion	Positive	10	71.4	%	8	53.3	%	0.315
Venous invasion	Positive	11	78.6	%	11	73.3	%	0.741

HSD: Heterogeneous score of diffusion-weighted image, SD: standard deviation, Non-dif: non-differentiated type such as poor, mucinous, and signet cell carcinoma.

**Table 3:** The correlation between heterogeneous score of DWI and tumor regression grade in pre-operative CRT cases.

	Tumor regression grade after CRT		
	1,2	3	p value
HSD before CRT	0.63 ± 0.14	0.46 ± 0.12	0.001
HSD after CRT	0.48 ± 0.18	0.39 ± 0.17	0.191

HSD: Heterogeneous score of diffusion-weighted image, CRT: chemoradiotherapy.

**Table 4:** The diagnostic accuracy for predicting of pathological staging and TRG of CRT.

		Cut-off	Sensitivity	Specificity	PPV	NPV	Accuracy
Primary resection group	T stage (T3 vs T2)	0.445<	54.5%	100.0%	100.0%	58.8%	65.5%
	N stage (N+ vs N-)	0.448<	66.7%	82.4%	72.7%	22.2%	86.2%
Pre-operative CRT group	TRG (1 vs 2,3)	0.695<	95.8%	53.8%	79.3%	12.5%	97.3%

PPV: positive predictive value, NPV: negative predictive value, CRT: chemoradiotherapy, TRG: tumor regression grade.

## Abbreviations

ADC: apparent diffusion coefficient map
AUC: area under the curve
CPT-11: irinotecan
CT: computed tomography
DWI: diffusion-weighted MRI
HSD: heterogeneous score of DWI
HSP: heterogeneous score of pathology
KPUM: Kyoto Prefectural University of Medicine
LLND: lateral lymph node dissection
LND: lymph node dissection
LNM: lymph node metastases
LRC: lower rectal cancer
MAX: maximum
MIN: minimum
MRI: magnetic resonance imaging
pCRT: pre-operative chemoradiotherapy followed by radical surgery cases
PR: primary resection cases
ROC: receiver operating characteristic
RT: radiotherapy
TME: total mesorectal excision
TRG: tumor regression grade
TS-1: tegafur, gimeracil, and oteracil potassium
TSR: tumor-stroma ratio
